# The *Conyza triloba* Extracts with High Chlorophyll Content and Free Radical Scavenging Activity Had Anticancer Activity in Cell Lines

**DOI:** 10.1155/2013/945638

**Published:** 2013-05-23

**Authors:** Wael M. El-Sayed, Warda A. Hussin, Ahmed A. Mahmoud, Mohamed A. AlFredan

**Affiliations:** ^1^Department of Biological Sciences and Department of Chemistry, Faculty of Science, King Faisal University, Al-Hofuf, Al-Ahsa 31982, Saudi Arabia; ^2^Department of Zoology, Faculty of Science, University of Ain Shams, Abbassia, Cairo 11566, Egypt; ^3^Department of Botany and Microbiology, Faculty of Science, Al-Azhar University, Cairo 11884, Egypt

## Abstract

The discovery of anticancer agents paradigm has been shifted to natural resources to overcome the toxicity of many synthetic agents at early clinical stages. In the present study, the antimutagenic, anticancer, phytochemistry, and free radical scavenging activities of five extracts of *Conyza triloba* were investigated. Extracts II (water : methanol), III (methylene chloride), and IV (methylene chloride : methanol) had the highest chlorophyll content and the highest superoxide scavenging, and metal chelating activities comparable to that of trolox. They also showed DPPH^•^ scavenging activities better than that of **α**-tocopherol. Virtually all extracts exerted a strong (>40% reduction) antimutagenic activity against sodium azide and benzopyrene. Extracts II, III, and IV showed a remarkable growth inhibition profile with GI_50_ of 0.07–0.87 **μ**g for Hepa1c1c7 and H4IIE1, A549, HT29, and PC3 cell lines and totally abated the growth of all cell lines, except for the breast cells, at 0.3–7.0 **μ**g. The present study found a strong correlation between the chlorophyll content of *Conyza* extracts and their DDPH scavenging, metal chelating, and in vitro cytotoxic and cytostatic activities most probably through triggering apoptosis. This study could offer a platform for future studies and help selecting the vital features that identify the extract with potential anticancer activities.

## 1. Introduction

Cancer is a continuing major health problem, and it is the second leading cause of death in the world. With the increasing level of carcinogens and mutagens in the environment, the search for new anticancer compounds has become crucial. Although many chemical anticancer agents are available, the side effects and the emergence of chemotherapy-resistant cancer cells among patients have urged the search for anticancer components from natural sources such as plants [1]. Using plants pharmacologically to treat cancer started early at the beginning of the last century. Silymarin and taxol showed outstanding success [2]. Other examples of naturally derived anticancer compounds are clinically in use, vincristine from *Vinca rosea*, etoposide from *Podophyllum peltatum*, and taxol from *Taxus brevifolia *[3]. According to WHO estimates, almost 80% of the population of developing countries rely on traditional medicines, mostly medicinal plants, for their primary health care needs. Demand for medicinal plants is increasing in both developing and developed countries due to the growing recognition of natural products being nonnarcotic, having less side-effects, and being available at affordable prices. 

The drug discovery of novel natural products with antitumor activity usually starts with a screening for plant extracts with significant antimutagenic/antioxidant activity and moving towards anticancer activity in cell lines. Since antimutagenic compounds are most probably to exert anticarcinogenic activity when challenged with carcinogens [4, 5], evaluation of antimutagenic activities of natural products appears to be necessary. This test as a prelude to any in vivo experimentation will save time and money in the screening pipeline. Studying the antimutagenicity of botanical extracts could provide the foundation for the utility of this test in selecting the lead extracts for long term and costly in vivo chemoprevention investigations and for separation and chemical structural elucidation of possible active compounds. 

The biological activity and chemical composition of *Conyza triloba* (family Asteraceae) have not been previously investigated, although the genera of these plants are safely edible for many years and medicinally used in other parts of the world. The plant is native to tropical and warm temperate regions throughout the world and is also found in North America and Eastern Asia. Furthermore, several extracts and chemical constituents isolated from this genus demonstrated a number of interesting biological activities such as antibacterial, anti-inflammatory, antitussive, antiulcer, and spasmolytic ones [6–8]. Previous chemical studies on *Conyza* species have led to the isolation of several types of compounds such as diterpenoids and flavonoids [6–8]. It is well documented that many flavonoids and quinones exert cytotoxic activity against several cancer cell lines in animals and humans including prostate [9], colon [10], breast [11], lung [12], and hepatic [13] cell lines.

Therefore, the aim of the present study was to investigate the potential antioxidant, antimutagenic, and anticarcinogenic activities of extracts with different polarities from *Conyza triloba* against mouse hepatic Hepa1C1C7, rat hepatic HepG2, human colon HT-29, human breast MCF-7, human lung A549, or human prostate PC3 cell lines. This study could offer aplatform for future studies and help selecting the key feature(s) of an extract that could most probably identify and/or predict the extract with potential cytotoxic and anticancer activities. This will reduce the time and cost of the screening process.

## 2. Materials and Methods

### 2.1. Chemicals

All reagents were purchased from Sigma (St. Louis, MO, USA) except where indicated in the specified methods. The aerial parts of* Conyza triloba* were collected from Dammam, district 71 (Saudi Arabia), in March 2011 and identified by a specialized taxonomist. A voucher specimen (CO-1-11) has been deposited in the Department of Biological Sciences, College of Science, King Faisal University, Saudi Arabia. The collected plant materials were stored in a dry and dark place at room temperature with a passive ventilation for 2 weeks. The dried plant materials were ground to powder using a plant grinder. Extraction of the chemical constituents of the plant materials by organic solvents according to polarity for biological screening and activities was performed for 48 h at room temperature according to the following extraction scheme; see Table 1.

The extracts were concentrated using a vacuum rotary evaporator. The extracts were sterilized by filtration through 0.2 *μ*m filter (Nalgene, Rochester, NY). The S9 mix consisted of a filter-sterilized NADPH (1.25 mM) and hepatic S9 fraction (4 mg protein/mL) prepared from male Sprague Dawley rats treated with a single dose (25 mg/kg, i.p. in corn oil) of Aroclor 1254 for three days. 

### 2.2. Cell Cultures and Bacteria

The *Salmonella typhimurium* TA1535, a histidine-mutant bacterial strain, was obtained from American Type Culture Collection (ATCC; Manassas, VA, USA) and used in antimutagenicity experiments. The cell lines used were hepatic mouse Hepa1C1C7 and rat H4IIE cells and human colon HT29, breast MCF7, lung A549, and prostate PC3. All cells, media, fetal bovine serum, DMSO, and trypsin-EDTA were obtained from ATCC (Manassas, VA, USA). Cell lines were seeded in 75 cm^2^ tissue culture flasks at 37°C in a humidified atmosphere (5% CO_2_), and the medium was renewed every two days.

### 2.3. Determination of Total Flavonoids

The total flavonoids was determined as described elsewhere [14]. Each plant extract (10 *μ*g) was mixed with 10% AlCl_3_·6H_2_O and 0.1 mL of 1 M potassium acetate, and the absorbance of reaction mixture was measured at 415 nm after 30 min. A standard curve for quercetin was established, and the data are expressed as mg quercetin equivalents/g dried plant materials. Assays were performed in triplicate. 

### 2.4. Determination of *β*-Carotene, Lycopene, and Chlorophylls a and b

*β*-Carotene, lycopene, and chlorophylls a and b were determined as described by Nagata and Yamashita [15]. A 100 mg of each extract was vigorously shaken with 10 mL of acetone-hexane mixture (4 : 6 v/v) for 1 min. The absorbance of the filtrate was measured at 453, 505, 645, and 663 nm. Contents of *β*-carotene, lycopene, and chlorophylls a and b were determined according to the following equations:

(1) lycopene (mg/100 mL)=−0.0458A663+0.204A645+0.372A505−0.0806A453,β-carotene (mg/100 mL)=0.216A663−1.22A645−0.304A505+0.452A453,chlorophyll  a (mg/100 mL)=0.999A663−0.0989A645,chlorophyll  b (mg/100 mL)=−0.328A663+1.77A645.

The results are expressed as means ± standard error of the mean (SEM). The values are expressed as mg/g extract. 

### 2.5. Determination of Total Antioxidant Activity

The antioxidant capacity of *Conyza* extracts was measured spectrophotometrically using a phosphomolybdenum method based on the reduction of Mo(VI) to Mo(V) and the subsequent formation of specific green phosphate/Mo(V) compounds [16]. A 0.3 mL aliquot of sample solution (1000 *μ*g/mL) was combined with 2.7 mL of the reagent solution (0.6 M sulfuric acid, 28 mM sodium phosphate, and 4 mM ammonium molybdate). The sample was capped and incubated in a boiling water bath at 95°C for 90 min. After cooling to room temperature, the absorbance was measured at 695 nm. Stock solutions of trolox were prepared in methanol just before use. The total antioxidant activity was expressed as equivalents of trolox (*μ*g trolox/g extract).

### 2.6. Peroxide Scavenging Activity

Peroxide scavenging activity was measured as described elsewhere [17]. Peroxide radicals were generated from mixing FeSO_4_ and H_2_O_2_. The reaction mixture contained 1 mL FeSO_4_ (1.5 mM), 0.7 mL H_2_O_2_ (6 mM), 0.3 mL sodium salicylate (20 mM), and either plant extract or trolox (100 *μ*g/mL). The mix was incubated for 1 h at 37°C, and the absorbance of the hydroxylated salicylate formed was measured at 562 nm. Consider the following:

(2) the peroxide scavenging activity(%)=[1−(A1−A2)A0]×100,

where *A*_0_ is the absorbance of the control (without extract or trolox), *A*_1_ is the absorbance including the extract or trolox, and *A*_2_ is the absorbance without sodium salicylate.

### 2.7. Superoxide Anion Scavenging Activity

Superoxide anion scavenging activity of the extracts was determined as described elsewhere [18]. Superoxide radicals were generated in phenazine methosulfate- (PMS-) nicotinamide adenine dinucleotide (NADH) systems and assayed by the reduction of nitroblue tetrazolium (NBT). One milliliter of extract or trolox (100 *μ*g/mL), 1.0 mL NBT solution (156 *μ*M NBT in 100 mM phosphate buffer, pH 7.4), and 1.0 mL NADH solution (468 *μ*M in 100 mM phosphate buffer, pH 7.4) were mixed. The reaction was started by adding 100 *μ*L of PMS solution (60 *μ*M PMS in 100 mM phosphate buffer, pH 7.4) to the mixture. The mixture was incubated at 25°C for 5 min, and the absorbance was measured at 560 nm against blank samples. The inhibition percentage of superoxide anion generation was calculated using the following formula:

(3) $$\begin{array}{c} \text{inhibition of superoxide anion}\left(\%\right)=\left[\frac{\left(A_{0}-A_{1}\right)}{A_{0}}\times 100\right], \end{array}$$

where *A*_0_ is the absorbance of control and *A*_1_ is the absorbance in presence of either extract or trolox.

### 2.8. Metal Chelating Activity

Plant extracts or standard antioxidants (0–600 *μ*g/mL) were added to 50 *μ*L of 2 mM FeCl_2_. The reaction was initiated by the addition of 5 mM ferrocene (0.2 mL), and the mixture was shaken vigorously at room temperature for 10 min [19]. The absorbance was measured at 562 nm. The metal chelating activity was calculated as follows:

(4) $$\begin{array}{c} \text{metal chelating effect}\left(\%\right)=\left[\frac{\left(A_{0}\;-A_{1}\right)}{A_{0}}\;\times 100\right], \end{array}$$

where *A*_0_ is the absorbance of control and *A*_1_ is the absorbance in presence of either extract or standards (trolox or *α*-tocopherol).

### 2.9. Free Radical Scavenging Activity

The free radical scavenging activity of *Conyza *extracts was evaluated using 2,2-diphenyl-1-picrylhydrazyl (DPPH^*∙*^) [20]. A 0.5 mL of 0.1 mM ethanolic solution of DPPH^•^ was added to 3.0 mL of extract or *α*-tocopherol (0–600 *μ*g/mL). The absorbance was measured at 517 nm after incubation for 30 min at room temperature. Consider the following:

(5) $$\begin{array}{c} \text{DPP}{\text{H}}^{•}\;\text{scavenging effect}\left(\%\right)=\left[\frac{\left(A_{0}\;-A_{1}\right)}{A_{0}}\times 100\right], \end{array}$$

where *A*_0_ is the absorbance of control and *A*_1_ is the absorbance in presence of either extract or *α*-tocopherol.

### 2.10. Cytotoxicity Assay on *Salmonella typhimurium* (TA1535)

The cytotoxicity assay was performed using the bacterial growth assay on nutrient agar plates [5]. The experiment was designed with conditions that mimic those of the revertant mutagenesis/antimutagenesis assay. In a preliminary experiment, a 10^−7^ dilution of TA1535 was shown to be the appropriate dilution; therefore, it was selected to proceed with. Briefly, a 100 *μ*L of 10^−7^ dilution of the overnight growing bacterial culture in Luria Broth (LB) medium was incubated with each plant extract (5, 10, or 20 mg/mL) and mixed with 2.5 mL of warm 0.6% top agar (NaCl/agar). The mix was then added to nutrient agar plates and the plates were incubated at 37°C for 24 hours. After the incubation period, colonies on triplicate plates were counted and compared to control plates containing no plant extracts. Concentrations investigated from hereafter were 1 and 5 mg for all extracts. 

### 2.11. Cytotoxicity of Plant Extracts in Combination with the Selected Mutagen

To rule out any possible toxic effect resulting from a combination of the mutagen used and plant extracts in the mutagenicity/antimutagenicity evaluation assays, the number of colony/plate evaluation was performed. A 100 *μ*L of 10^−7^ dilution of overnight growing *S. typhimurium* TA1535 was incubated with each extract at 37°C for 30 minutes on a shaking incubator in the presence of 2 *μ*g/plate of NaN_3_ or 20 *μ*M of B[a]P and S9 mix in 400 *μ*L of phosphate buffer. The mix was then added to agar plates and incubated at 37°C, and the total viable bacterial count on triplicate plates was recorded after 24 hours [21]. Control experiments were carried out simultaneously, and the S9 mix alone had no effect on the bacterial viability. 

### 2.12. Mutagenicity Assays

The reverse bacterial mutation assay was performed for screening the mutagenic potential of the plant extracts as described elsewhere [4]. Briefly, a 100 *μ*L of 15-hour growing *S. typhimurium* TA1535 (1 × 10^9^ CFU/mL) in LB medium was preincubated with each extract well below the toxic concentration at 37°C for 30 minutes in the presence of S9 mix. The incubate was then added to top agar containing 50 *μ*M of histidine/biotin and poured on to minimal glucose agar (MGA) plates. Revertant colonies were counted after incubation at 37°C for 36–48 hours. Spontaneous revertant colonies arising on plates containing neither mutagens nor extracts were also counted. Revertant colonies seen with 20 *μ*M B[a]P with S9 mix were performed as a positive control. All assays were performed in triplicates.

### 2.13. Antimutagenicity Assays

Antimutagenic activity of *Conyza triloba* extracts against NaN_3_ or B[a]P was determined under preexposure and coexposure assays by a modified method of Maron and Ames [4]. Under preexposure conditions, each extract was incubated with *S. typhimurium* TA1535 at 37°C for 30 minutes before the addition of B[a]P (20 *μ*M) or NaN_3_ (2 *μ*g/plate). Coexposure assays were performed by incubating the bacteria at 37°C for 30 minutes with the mutant and each extract prior to plating on MGA plates. In all assays, positive and negative controls were performed. All antimutagenesis determinations were performed in triplicates. Revertant colonies were counted after 36–48 hours of incubation, and the antimutagenic potential of the tested extracts was expressed as a percentage of reduction in mutagenicity [22] and calculated according to the following equation:

(6) %  reduction in mutagenicity=([Rm−Rs]−[Ra−Rs][Rm−Rs])×100,

where *R*_*m*_ is the number of revertants/plate in the presence of mutagen, *R*_*s*_ is the number of spontaneous revertants/plate, and *R*_*a*_ is the number revertants/plate in the presence of plant extract.

A 20% or less reduction means no antimutagenic activity, 20%–40% reduction means a moderate activity, and 40% or more reduction means a strong antimutagenic activity. 

The mutant frequency or mutation rate was then calculated from the mutant colonies/viable colonies for both exposure conditions for the mutagens investigated. All procedures were approved by the University of King Faisal Committee of Scientific Research Ethics.

### 2.14. Determination of In Vitro Anticancer Activity

The anticancer activity was determined as described elsewhere [23]. The cell lines were grown in the suitable medium. The cells were inoculated into 96-well plates at plating densities of ~5,000 cells/well, and four wells were used for each treatment. Since the extracts are colored, control wells (extract control) were made for every extract concentration used without cells. Three independent experiments were performed. The plates were then incubated at 37°C and 5% CO_2_ for 24 h prior to addition of plant extracts. After 24 h, two plates of each cell line were fixed with trichloroacetic acid (TCA), to measure the cell population for each cell line at the time of extract addition (*T*_0_). Aliquots of 100 *μ*L of different extract dilutions in DMSO vehicle (0.1%, final concentration that does not affect cell viability) were sterilized by filtration and added to the appropriate wells of fresh plates. The plates were further incubated for an additional 48 h. The assay was terminated by the addition of cold TCA. Cells were fixed *in situ* by the addition of 50 *μ*L of cold 50% (w/v) TCA (final concentration, 10% TCA) and incubated for 60 minutes at 4°C. The supernatant was discarded, and the plates were washed three times with tap water and air dried. Sulforhodamine B (SRB) solution (100 *μ*L) at 0.4% (w/v) in 1% acetic acid was added to each well, and plates were incubated for 10 minutes at room temperature. After staining, unbound dye was removed by washing three times with 1% acetic acid and the plates were air dried. Bound stain is then solubilized with 10 mM Trizma base, and the absorbance was read at a wavelength of 515 nm. 

Percentage growth inhibition was calculated using the following formula:

(7) [T−T0C−T0]×100  for concentrations for which  T   ≥T0,[T−T0T0]×100  for  concentrations  for  which  T   <T0.

*T*_0_ or time zero represents a measurement of the cell population for each cell line at the time of extract addition, *C* is the control growth, and *T* is the test growth at different concentrations of *Conyza* extracts after incubation. Three dose response parameters were calculated for each experimental agent. Growth inhibition of 50% (GI_50_) was calculated from [(*T* − *T*_0_)/(*C* − *T*_0_)] × 100 = 50, which is the extract concentration resulting in a 50% reduction in the net protein increase (as measured by SRB staining) in control cells during the incubation. The extract concentration resulting in total growth inhibition (TGI) is calculated from *T*_*i*_ = *T*_*z*_. The LC50 (concentration of extract resulting in a 50% reduction in the measured protein at the end of the extract treatment as compared to that at the beginning) indicating a net loss of cells following treatment is calculated from [(*T*_*i*_ − *T*_*z*_)/*T*_*z*_] × 100 = −50.

### 2.15. Statistical Analysis of Data

Statistical analyses were performed using ANOVA, followed by Fisher's protected least significant difference multiple range test. Differences were considered significant at *P* < 0.05.

## 3. Results and Discussion

### 3.1. Phytochemistry and Radical Scavenging Activity

We have used five solvents with different polarities. Extract I (H_2_O extract) presumably has the highly polar compounds such as sugars, glucosides, and aglycones, extracts II (methanol : water), III (methylene chloride), and IV (methylene chloride : methanol) should contain the intermediate polar flavonoids and glycosides, while extract V (hexane extract) would contain the nonpolar compounds. 

**Figure 1:**
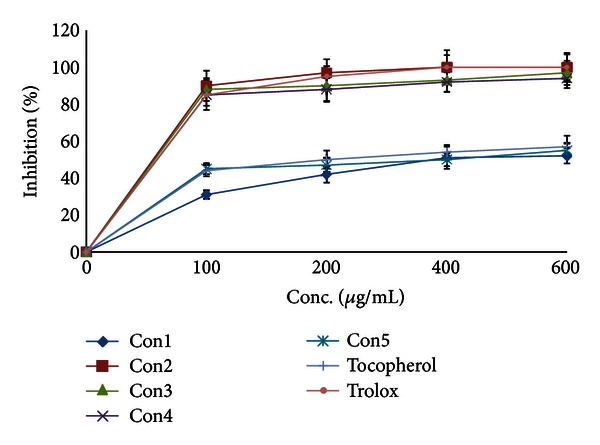
Metal chelating activity of *Conyza triloba* extracts.

**Figure 2:**
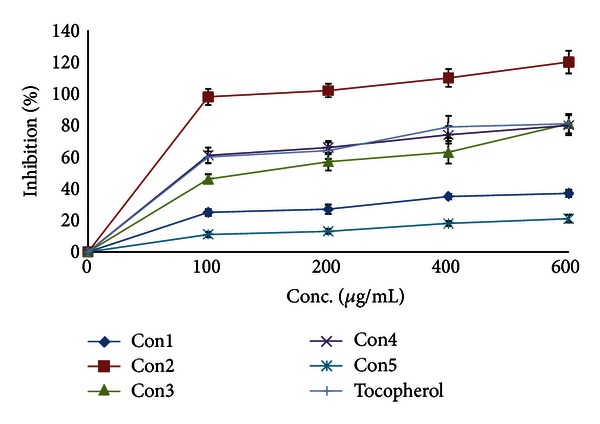
Free radical scavenging activity of *Conyza triloba* extracts.

As expected from the solvent polarity, only extract II had a high content of total flavonoids equivalent to ~94 mg quercetin/g. Extract IV had a moderate content of total flavonoids equivalent to ~15 mg quercetin/g (Table 2), while extract V had almost no flavonoids content. *Conyza* extracts were all poor in lycopene and *β*-carotene contents (~11–29 and 30–40 mg/g, resp.). Extracts II, III, and IV had the highest chlorophylls a and b content of ~10%, 8%, and 15% and 8%, 7%, and 6% (w/w), respectively (Table 2). The highest total antioxidant activity was shown by extract II followed by I and IV (Table 3). This antioxidant activity could be attributed to the total flavonoid content. However, extract I with somewhat low flavonoid content had high antioxidant and peroxide scavenging activities (Table 3). Therefore, there must be other highly polar components responsible for these activities. Almost all *Conyza* extracts had peroxide and superoxide anion scavenging activities more than trolox (Table 3). Extracts II, III, and IV had the highest superoxide scavenging activity (Table 3) and a metal chelating activity comparable to that of trolox (Figure 1). This metal chelating activity was a concentration dependent. Extracts I and V had a lower activity and almost comparable to that of *α*-tocopherol (Figure 1). The superoxide anion can form more reactive free radicals resulting in disrupting the cellular structure [24]. DPPH is somewhat a stable free radical that could be reduced forming a diamagnetic molecule. Extracts III and IV had a free radical (DPPH^*∙*^) scavenging activity similar to *α*-tocopherol, while extract II showed a much better scavenging profile. The flavonoids and phenolic compounds present in these extracts could be responsible for this scavenging activity because of their electron and/or hydrogen donating capability. Extracts I and V had low scavenging effects on DPPH^•^ (Figure 2). Many studies showed that reactive oxygen species (ROSs) take part at least in one stage in the development of cancer [1–3, 24]. The ROSs cause destructive and irreversible damage to the cellular components such as lipids, proteins, and DNA. The continuous accumulation of damage to the cells induces diseases such as cancer and aging. The extracts of some *Conyza* species rapidly scavenged DPPH [25]. The inhibition of free radical generation can serve as a facile model for evaluating the activity of anticancer agents. There is a strong correlation between the high chlorophylls a and b content in *Conyza* extracts II, III, and IV and their superoxide anion and DPPH^•^ free radical scavenging activities and iron metal chelation capacity. The prudent application of this prediction module could significantly influence the screening process of natural products of plant origin. Therefore, we believe that methanol : water extract had some compounds that deserve further studies. This extract is now subjected to intensive fractionation and identification of the active chemicals. 

### 3.2. Effect of Plant Extracts on the *Salmonella typhimurium* Viability

*Conyza* extracts at 5 and 10 mg/mL had no significant effect on the viability of *Salmonella typhimurium* TA1535. All extracts at 20 mg/mL resulted in significant reductions in the bacterial viability. Therefore, we decided to proceed with 1 and 5 mg/mL well below any concentration that could compromise the bacterial viability. The effect of a combination of NaN_3_ (2 *μ*g/plate) or B[a]P (20 *μ*M) with plant extracts (1 or 5 mg/mL) on the viability was investigated at conditions that mimic those that will be used in the antimutagenicity studies. This prerequisite study is essential to rule out any interference from a possible overt toxicity from the concurrent exposure of the bacteria to both the mutagen and extract and to validate the antimutagenicity results. The combination of either mutagens with the plant extracts had no effect on the bacterial viability. 

### 3.3. Mutagenic/Antimutagenic Activity of *Conyza* Extracts

The antimutagenic properties of the botanical extracts were investigated against sodium azide (NaN_3_) and benzo[a]pyrene (B[a]P) using the *Salmonella* reverse bacterial assay. Mutation is an early key step in the cancer development, and the *Salmonella* reverse assay is of great importance in the detection of antimutagenic/anticarcinogenic agents in the pipeline of drug discovery and development [21]. This antimutagenic study was a useful surrogate marker that guided the selection of potential extracts for further screening process in cell lines and animal models. Two mutagens were selected for antimutagenic studies, NaN_3_ and B[a]P. Both act in different pathways; the azide is a direct mutagen without any metabolic activation, and the B[a]P is a known polycyclic aromatic hydrocarbon that needs a metabolic activation by P4501A1 [26]. *S. typhimurium* TA1535 strain contains the base-pair substitution mutation *hisG46* [27], which is known to be more responsive to sodium azide than other direct mutagens [4]. On examining the potential mutagenic activity of extracts, all extracts were not mutagenic, that is, did not cause a doubling in the number of colonies over the spontaneous number in presence of S9 mix. The potential antimutagenic activity of *Conyza* extracts was evaluated using a modified Ames assay in preexposure and coexposure treatments. With the exception of extract I at both concentrations used and extract II at the low concentration under coexposure regimen, all extracts exerted a strong (>40% reduction) antimutagenic activity against NaN_3_ (Table 4). The reduction in the number of colonies was concentration dependent for extracts I, II, and IV, while extracts III and V resulted in almost the same antimutagenic activity regardless of the concentration. This could result from a saturation effect. The type of exposure had no effect on the antimutagenic activity of extracts III, IV, and V. Under preexposure conditions, extracts I and II showed a much better antimutagenic activity (Table 4). The same pattern was shown in mutation frequency where the effective antimutagenic plant extracts reduced the mutation frequency by 28%–96% (Table 6). All plant extracts had a strong (>40%) antimutagenic activity against B[a]P. Only extracts I and V showed concentration dependency under preexposure conditions only, while extracts II, III, and IV showed the same antimutagenic activity at both concentrations used, a possible plateau effect. The antimutagenic activity of extracts was more evident under preexposure conditions in which the extracts were incubated with the bacteria for 30 min prior to the addition of B[a]P (Table 5). All extracts reduced the mutation frequency by 49%–86% (Table 7). The antimutagenic activities of *Conyza* could be attributed to the direct binding and protection of DNA from the electrophilic mutagens or metabolites [28]. Another mechanism could be the elevation in the antioxidants milieu of the cells, thus promoting the DNA repair systems [29]. The high total antioxidant, metal chelating, peroxide, and superoxide scavenging activities support this possibility. A third mechanism could be the direct interaction with the mutagens or their metabolites preventing their damaging effects [30]. Since B[a]P is activated by CYP1A1, one of the possible mechanisms would be the inhibition of this bioactivation [21].

### 3.4. Anticancer Activity

**Figure 3:**
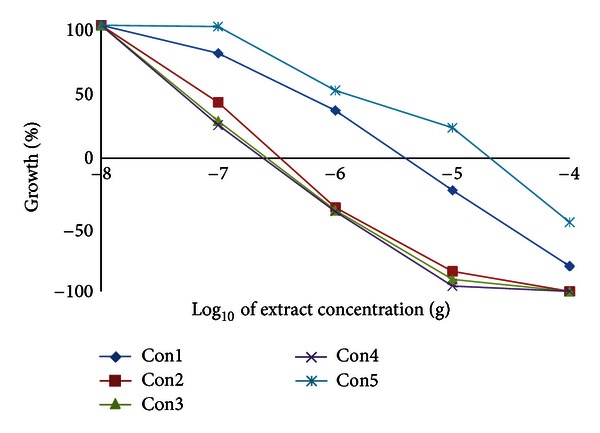
Effect of *Conyza triloba* extracts on Hepa1C1C7 growth.

**Figure 4:**
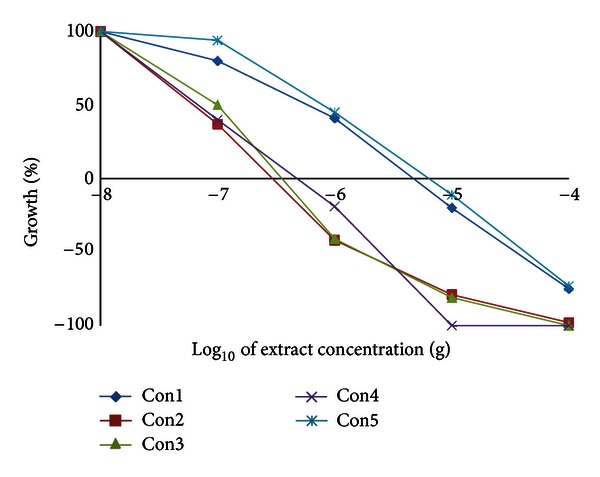
Effect of *Conyza triloba* extracts on H4IIE1 growth.

**Figure 5:**
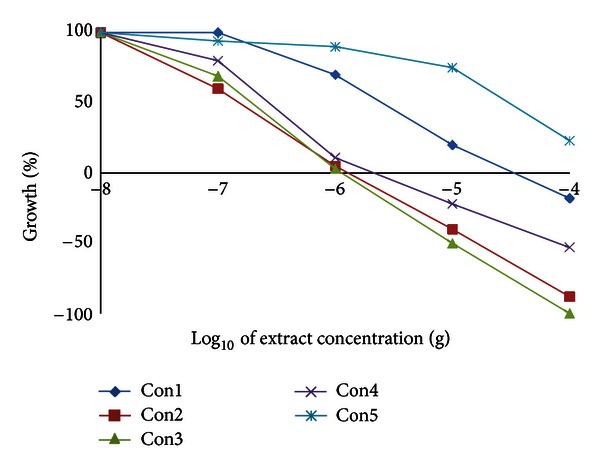
Effect of *Conyza triloba* extracts on A549 growth.

**Figure 6:**
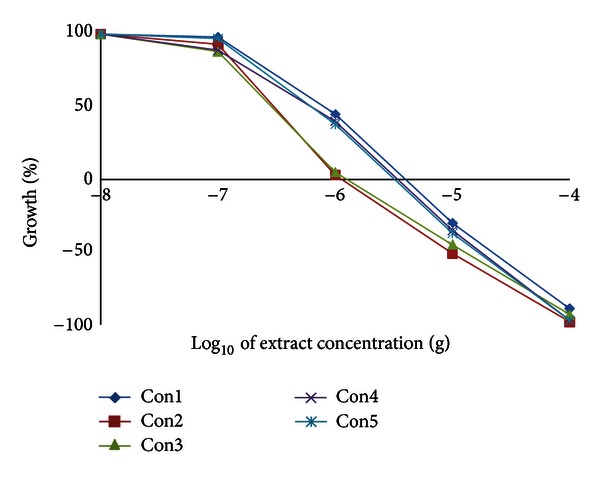
Effect of *Conyza triloba* extracts on HT29 growth.

**Figure 7:**
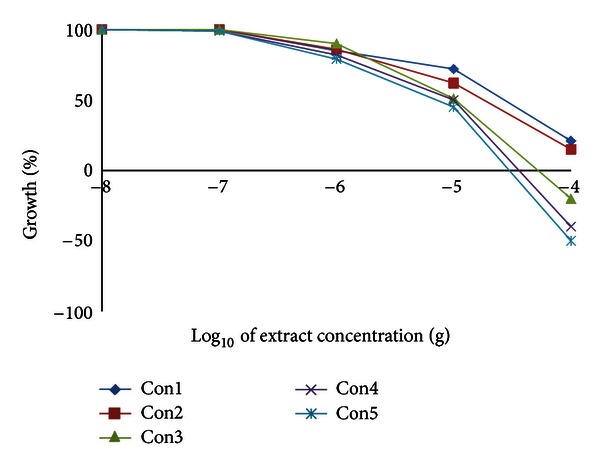
Effect of *Conyza triloba* extracts on MCF7 growth.

**Figure 8:**
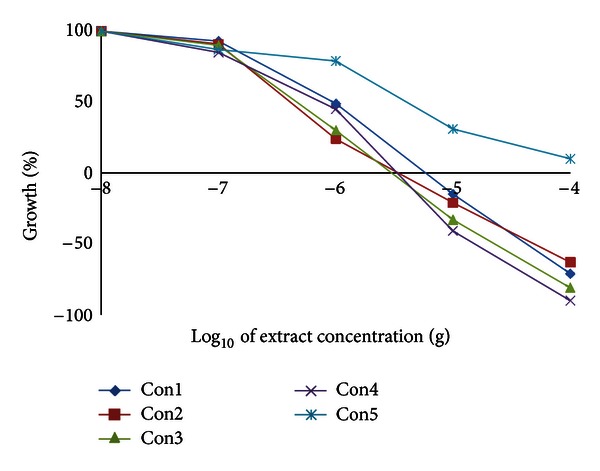
Effect of *Conyza triloba* extracts on PC3 growth.

We have examined the cytotoxic activity of the *Conyza* extracts in 6 cell lines representing 5 organs; hepatic mouse Hepa1C1C7 and rat H4IIE cells and human colon HT29, breast MCF7, lung A549, and prostate PC3. In hepatic (Hepa1C1C7 and H4IIE1) and lung (A549) cells, the extracts split into two groups. The highly active group included extracts II, III, and IV, and the less active group included extracts I and V (Figures 3, 4, and 5 and Table 8). Extracts II, III, and IV showed a remarkable growth inhibition profile on lung and liver cells investigated with GI_50_ of 0.07–0.1 *μ*g for both hepatic cell lines investigated and ~0.3 *μ*g for lung cells. The colon cell line was the most prone, and the GI_50_ recorded for all extracts (Table 8(a)) was about 0.5–1.0 *μ*g with extracts II and III being the most effective (Figure 6). The breast cell line was the most resilient, and the GI_50_ values recorded for all extracts were ~8.5–44.0 *μ*g (Table 8(a) and Figure 7). Extracts I, II, III, and IV were the most effective against prostate PC3 cells with GI_50_ of 0.6–1 *μ*g, while extract V was devoid of any effect on these prostate cells with GI_50_ of 6.0 *μ*g (Table 8(a) and Figure 8). The data in Table 8(b) show that these three extracts (II, III, and IV) totally abated the growth of all cell lines at concentrations of as low as 0.3–0.7 *μ*g for liver cells, ~2.0–3.0 *μ*g for colon and lung cells, and ~7.0 *μ*g for prostate cells. Extract IV achieved a total growth inhibition of colon cells at a relatively higher concentration (~6 *μ*g) than those of extracts II and III. All extracts needed very high concentrations (47.0–375.0 *μ*g) to achieve the total growth inhibition of breast cell line (MCF7). Similar results were shown in Table 8(c) when calculating the 50% lethality (LC_50_). Natural products could exert their anticancer activities through various mechanisms such as inhibition of carcinogens, modulation of cancer cell signaling, and triggering apoptosis and cell cycle arrest. Many of these effects are related to their antioxidant activities [31].

Although the screening of anticancer activity of natural products in cell lines is cost-effective and time saving, but it lacks some key advantages of in vivo assays. Therefore, the absolute in vitro activity is not always proportional to the clinical efficacy in vivo. On the other hand, some inactive extracts in vitro could exert their effects on immune system or need biotransformation and this could result in discarding some potential extracts from further in vivo studies. The present study through many parameters investigated found a correlation between the chlorophyll content of three *Conyza* extracts containing intermediate polar compounds and their DDPH scavenging activity, metal chelating activity, and in vitro cytotoxic and cytostatic activities in lung, colon, liver, and prostate cell lines probably through triggering apoptosis. These extracts showed substantial superoxide anion and DPPH^•^ free radical scavenging activities and a significant iron metal chelation capacity. However, the reverse bacterial mutation assay could not differentiate the extracts or correlate well with the in vitro anticancer activity since almost all extracts showed antimutagenic activities under the experimental conditions used. In agreement with our findings, a single previous study found that a chlorophyll-derived compound isolated from plant leaves had significant anticancer activity in human cell lines [32]. Some extracts from a different *Conyza* species scavenged the DPPH radicals [25] and exhibited the highest cytotoxic activity in lung and prostate cell lines [33]. This study could offer a platform for future studies and help selecting the vital features of an extract that identify the extract with potential anticancer activities. This will reduce the time and cost of the screening process. Further studies are needed to confirm the liability of the results in animal models and identify the main active components of *Conyza* extracts II, III, and IV. 

## Figures and Tables

**Table 1:** 

Sample code	Extraction solvent
Conyza-1 (I)	H_2_O 100%
Conyza-2 (II)	H_2_O : MeOH (1 : 1)
Conyza-3 (III)	CH_2_Cl_2_ 100%
Conyza-4 (VI)	CH_2_Cl_2_ : MeOH (1 : 1)
Conyza-5 (V)	*n*-Hexane 100%

**Table 2:** Total flavonoids, lycopene, *β*-carotene, and chlorophylls a and b content of different *Conyza* extracts.

Extract	Total flavonoids (mg quercetin)^a^	Lycopene (mg/g)^a^	*β*-carotene (mg/g)^a^	Chlorophyll a (mg/g)^a^	Chlorophyll b (mg/g)^a^
I	6.7 ± 0.6	23.3 ± 1.4	40.7 ± 2.8	46.0 ± 1.7	43.7 ± 2.9
II	93.3 ± 2.5	28.7 ± 2.1	41.3 ± 3.0	104.3 ± 8.9	80.3 ± 5.8
III	3.7 ± 0.2	25.0 ± 2.2	35.0 ± 3.1	80.7 ± 5.5	65.7 ± 4.6
IV	14.7 ± 1.4	28.7 ± 1.8	32.3 ± 1.2	152.7 ± 6.7	63.0 ± 6.4
V	0.3 ± 0.0	11.3 ± 1.2	29.0 ± 2.0	65.7 ± 6.6	51.3 ± 3.2

^a^The data are expressed as means ± SEM. All assays were performed in triplicates.

**Table 3:** Total antioxidant, peroxide scavenging, and superoxide scavenging activities of various *Conyza* extracts.

Treatment	Antioxidant activity ^a,b^	Peroxide scavenging activity (%)^a^	Superoxide scavenging activity (%)^a^
I	91.3 ± 1.4	67.3 ± 3.1	65.0 ± 3.5
II	132.7 ± 2.2	49.7 ± 2.0	96.7 ± 4.7
III	4.7 ± 0.9	42.0 ± 1.8	84.0 ± 4.6
IV	67.7 ± 2.1	45.7 ± 4.0	81.0 ± 5.2
V	1.3 ± 0.1	28.7 ± 2.2	62.0 ± 5.5
Trolox	192.3 ± 3.5	32.3 ± 2.9	61.7 ± 3.7

^a^The data are expressed as means ± SEM; *n* = 3.

^b^The activity is expressed as equivalent of trolox (*μ*g trolox/g extract).

**Table 4:** Determination of antimutagenic activity of *Conyza* extracts in *Salmonella typhimurium* TA1535 against sodium azide (NaN_3_; 2 *μ*g/plate).

Treatment	NaN_3_ mutagenicity at *Conyza* extract concentrations (revertant colonies/plate; mean ± SEM (% reduction in NaN_3_ mutagenicity))			
	Preexposure*		Coexposure*	
	1 mg/mL	5 mg/mL	1 mg/mL	5 mg/mL
NaN_3_ (2 *μ*g/plate)	290.3 ± 17.1 (0)			
I	179.0 ± 10.5 (41)^a^	31.3 ± 5.2 (95)^a,b^	286.0 ± 18.0 (4)	215.0 ± 18.1 (28)^a,b^
II	129.3 ± 4.2 (59)^a^	11.3 ± 1.9 (102)^a,b^	291.0 ± 21.5 (0)	24.0 ± 4.7 (97)^a,b^
III	22.3 ± 2.9 (98)^a^	13.3 ± 1.5 (101)^a^	14.3 ± 2.7 (101)^a^	10.0 ± 1.7 (103)^a^
IV	64.0 ± 5.7 (83)^a^	18.7 ± 3.7 (99)^a,b^	74.0 ± 4.1 (79)^a^	20.7 ± 1.2 (99)^a,b^
V	10.7 ± 1.2 (102)^a^	13.7 ± 1.5 (101)^a^	58.7 ± 8.0 (85)^a^	31.3 ± 6.1 (95)^a^

*Plant extracts were incubated with bacteria either 30 minutes before NaN_3_ (Preexposure) or incubated with the bacteria and NaN_3_ (coexposure). The spontaneous revertant colonies were 17.0 ± 2.1. ^a^Significant (*P* < 0.05) reduction (% of inhibition of mutagenicity indicated in parentheses) from revertant colonies seen with NaN_3_. ^b^Significant difference (*P* < 0.05) between plant extract concentrations.

**Table 5:** Determination of anti-mutagenic activity of plant extracts in *Salmonella typhimurium* TA1535 against benzo[a]pyrene (B[a]P; 20 *μ*M) in presence of S9 mix.

Treatment	B[a]P mutagenicity at plant extract concentrations (revertant colonies/plate; mean ± SEM (% reduction in B[a]Pmutagenicity))			
	Preexposure*		Coexposure*	
	1 mg/mL	5 mg/mL	1 mg/mL	5 mg/mL
B[a]P	324.3 ± 21.5			
I	147.7 ± 5.9 (63)^a^	109.0 ± 10.2 (77)^a,b^	160.7 ± 10.5 (59)^a^	150.0 ± 7.1 (62)^a^
II	63.7 ± 11.1 (93)^a^	52.7 ± 6.1 (97)^a^	97.7 ± 6.7 (81)^a^	93.7 ± 9.2 (83)^a^
III	55.0 ± 4.7 (96)^a^	63.3 ± 7.5 (93)^a^	98.0 ± 7.8 (81)^a^	91.7 ± 4.7 (83)^a^
IV	54.0 ± 2.9 (97)^a^	47.3 ± 3.7 (99)^a^	95.3 ± 3.5 (82)^a^	90.7 ± 9.3 (84)^a^
V	92.3 ± 4.1 (83)^a^	58.3 ± 7.3 (95)^a,b^	111.7 ± 5.8 (76)^a^	105.3 ± 8.7 (78)^a^

*Plant extracts were incubated with bacteria either 30 minutes before B[a]P (preexposure) or incubated with the bacteria and B[a]P (coexposure). The spontaneous revertant colonies were 45.0 ± 4.9. ^a^Significant (*P* < 0.05) reduction (% of inhibition of mutagenicity indicated in parentheses) from revertant colonies seen with B[a]P. ^b^Significant difference (*P* < 0.05) between plant extract concentrations.

**Table 6:** Effects of plant extracts on sodium azide (NaN_3_) mutant frequency.

Treatment	Mutant frequency and % of NaN_3_^#^			
	Preexposure*		Coexposure*	
	1 mg/mL	5 mg/mL	1 mg/mL	5 mg/mL
NaN_3_	6.70 (100)			
I	4.80 (72)^a^	0.85 (13)^a^	7.67 (114)	5.81 (87)
II	3.13 (47)^a^	0.28 (4)^a^	7.05 (105)	0.60 (9)^a^
III	0.53 (8)^a^	0.32 (5)^a^	0.34 (5)^a^	0.24 (4)^a^
IV	1.53 (23)^a^	0.45 (7)^a^	1.77 (26)^a^	0.49 (7)^a^
V	0.24 (4)^a^	0.34 (5)^a^	1.33 (20)^a^	0.77 (11)^a^

^#^Calculated from mutant colonies (Table 4)/viable colonies. *Plant extracts were incubated with bacteria either 30 minutes before NaN_3_ (preexposure) or incubated with the bacteria and NaN_3_ (coexposure). ^a^Significant difference (*P* < 0.05) from NaN_3_.

**Table 7:** Effects of plant extracts on benzo[a]pyrene (B[a]P) mutant frequency.

Treatment	Mutant frequency and % of B[a]P^#^			
	Preexposure*		Coexposure*	
	1 mg/mL	5 mg/mL	1 mg/mL	5 mg/mL
B[a]P	9.54 (100)			
I	4.44 (46)^a^	2.82 (30)^a^	4.83 (51)^a^	3.88 (41)^a^
II	1.95 (20)^a^	1.35 (14)^a^	2.99 (31)^a^	2.40 (25)^a^
III	1.83 (19)^a^	1.57 (16)^a^	3.27 (34)^a^	2.28 (24)^a^
IV	1.59 (17)^a^	1.36 (14)^a^	2.80 (29)^a^	2.61 (27)^a^
V	2.41 (25)^a^	2.27 (24)^a^	2.92 (31)^a^	4.10 (43)^a^

^#^Calculated from mutant colonies (Table 5)/viable colonies. *Plant extracts were incubated with bacteria either 30 minutes before B[a]P (preexposure) or incubated with the bacteria and B[a]P (coexposure). ^a^Significant difference (*P* < 0.05) from B[a]P.

**Table:** (a)

Extract	Potency of extracts (*μ*g) in cell lines (mean ± SEM); *n* = 3					
	HT29	A549	PC3	MCF7	Hepa1C1C7	H4IIE1
I	0.93 ± 0.10	4.07 ± 0.31	1.00 ± 0.05	43.77 ± 3.02	0.70 ± 0.04	0.93 ± 0.04
II	0.47 ± 0.05	0.17 ± 0.01	0.60 ± 0.02	28.13 ± 1.87	0.10 ± 0.01	0.07 ± 0.01
III	0.47 ± 0.02	0.27 ± 0.01	0.67 ± 0.03	10.00 ± 0.79	0.07 ± 0.02	0.10 ± 0.01
IV	0.77 ± 0.04	0.43 ± 0.03	0.87 ± 0.05	10.00 ± 0.88	0.07 ± 0.01	0.07 ± 0.02
V	0.77 ± 0.07	48.43 ± 4.00	6.10 ± 0.53	8.43 ± 0.51	1.00 ± 0.07	0.77 ± 0.06

**Table:** (b)

Extract	Potency of extracts (*μ*g) in cell lines (mean ± SEM); *n* = 3					
	HT29	A549	PC3	MCF7	Hepa1C1C7	H4IIE1
I	6.3 ± 0.51	50.0 ± 6.21	7.7 ± 0.44	375.0 ± 29.12^ a^	6.0 ± 0.41	7.0 ± 0.91
II	1.7 ± 0.01	1.9 ± 0.07	7.0 ± 0.52	281.3 ± 31.04^ a^	0.7 ± 0.34	0.3 ± 0.02
III	2.0 ± 0.09	1.6 ± 0.02	6.7 ± 0.47	71.7 ± 5.55	0.3 ± 0.22	0.7 ± 0.01
IV	5.7 ± 0.49	3.3 ± 0.02	7.0 ± 0.34	56.3 ± 4.67	0.3 ± 0.24	0.7 ± 0.03
V	5.3 ± 0.61	437.0 ± 41.11^a^	478.7 ± 39.29^ a^	46.7 ± 6.00	34.3 ± 4.01	8.0 ± 1.01

**Table:** (c)

Extract	Potency of extracts (*μ*g) in cell lines (mean ± SEM), *n* = 3					
	HT29	A549	PC3	MCF7	Hepa1C1C7	H4IIE1
I	37.3 ± 4.5	845.0 ± 98.4^a^	64.0 ± 5.9	>1000^a^	48.7 ± 5.3	56.3 ± 6.1
II	10.0 ± 2.1	21.7 ± 3.4	71.7 ± 6.4	>1000^a^	3.0 ± 2.4	2.0 ± 0.5
III	19.0 ± 1.8	10.0 ± 1.5	37.7 ± 2.8	375.0 ± 40.1^a^	2.3 ± 1.6	2.0 ± 0.4
IV	31.3 ± 2.5	91.0 ± 8.7	18.7 ± 3.3	193.7 ± 21.9	1.7 ± 0.7	4.0 ± 0.3
V	25.7 ± 2.7	>1000^a^	>1000^a^	100.0 ± 11.1	130.0 ± 9.9^a^	65.7 ± 8.0

^a^Extrapolated from dose-response curve. HT29 (human colon), A549 (human lung), PC3 (human prostate), MCF7 (human breast), Hepa1C1C7 (murine liver), and H4IIE1 (rat liver). GI50 is the concentration of an extract (*μ*g) that causes 50% growth inhibition. TGI is the concentration of an extract (*μ*g) that yields no net growth over the course of the assay. LC50 is the concentration of an extract (*μ*g) that kills 50% of the cells that were present at the time of the addition of the extract.
